# Monoclonal Antibodies Against Myeloid Leukemia Cells: Current Knowledge and Future Directions

**DOI:** 10.3390/ijms26104571

**Published:** 2025-05-10

**Authors:** Daniela Damiani, Mario Tiribelli

**Affiliations:** 1 Division of Hematology and Stem Cell Transplantation, Udine University Hospital, 33100 Udine, Italy; mario.tiribelli@uniud.it; 2 Department of Medicine, University of Udine, 33100 Udine, Italy

**Keywords:** acute myeloid leukemia, chemotherapy, relapse, prognosis, leukemia stem cells, monoclonal antibodies

## Abstract

Monoclonal antibodies targeting specific cell surface antigens have emerged as a promising therapeutic approach for acute myeloid leukemia (AML), thus widening the treatment landscape of this heinous disease. These antibodies have been designed to selectively target and eliminate leukemic cells while limiting damage to the normal hematopoietic counterpart. Among the potential targets on AML cells, CD33, CD123, and CD47 have shown the major potential in preclinical and clinical trials. Additionally, conjugation of monoclonal antibodies with cytotoxic agents has further enhanced their therapeutic efficacy. Nonetheless, challenges such as antigen heterogeneity, resistance mechanisms, and the immunosuppressive tumor microenvironment remain significant barriers to achieving durable remission in AML patients. This review explores the mechanisms of action, current clinical developments, and ongoing trials into the role of monoclonal antibodies in AML, highlighting their potential to improve clinical outcomes when used alone or in combination with conventional therapies, making them thus able to become, in the near future, a cornerstone in the treatment of AML.

## 1. Introduction

Acute myeloid leukemia (AML) is a genetically heterogeneous disease defined by clonal expansion and differentiation arrest of myeloid progenitor cells, which result in peripheral cytopenia and increased risk of life-threatening infections and hemorrhages [1]. AML accounts for 10–20% of leukemia cases in western counties and its incidence is increasing as a consequence of prolonged general life expectancy, of environmental and occupational exposure, and of higher survival rates after cancer treatment [2,3]. Over the past five decades, the limited therapeutic options made the old “3 + 7” anthracycline + cytarabine combination regimen the standard of care for induction therapy in all non-promyelocytic AMLs, irrespective of disease characteristics and patient age [4,5]. Its limited efficacy has been reported in many clinical trials, both in younger patients, in which long term survival rate is 40% or less [6], and in patients fit for intensive therapy but over 60 years, whose 5-year overall survival (OS) rate is 10% or less [7]. The efficacy of 3 + 7 was only slightly improved over time by anticipating, in induction, the use of high-dose cytarabine [8,9], traditionally used only in consolidation courses [10,11]. In patients younger than 65 years, most of the trials investigating the role of high-dose cytarabine in induction demonstrated longer relapse-free survival (RFS) [8,12,13,14] and, in some cases, also an advantage in complete remission (CR) rate [13,14]. However, it must be underlined that RFS was less than 50%, progressively shortening with increasing age. Better results were obtained through the addition of nucleoside analogs (fludarabine or cladribine), increasing long-term RFS to 66%, at the cost of increased myelosuppression-associated side effects [10,15], and through the change of anthracycline, idarubicin, or mitoxantrone vs. daunorubicin [16,17].

As such, allogenic hematopoietic cell transplantation (HCT) remains the only potentially curative option [18,19,20]. Response and survival rate are particularly low in secondary AML, an adverse subset irrespective of age that accounts for about 15–20% of AMLs and, in older (i.e., 75–80 years) and/or unfit patients, represents more than 40% of all cases and that were, for a long time, deemed as candidates for supportive care.

The exponential advances in molecular diagnosis in the last two decades have not only allowed for a better knowledge of AML pathophysiology, but also identified recurrent molecular alterations with prognostic significance, especially in the subset with normal/intermediate cytogenetics [21]. The advent of next generation sequencing (NGS), able to detect recurrent somatic mutations in 90% of patients with AML [22,23], further refined the prognostic scores, identified many targetable alterations, and helped the selection of treatment, definitively closing the era of “one-size-fits-all” and moving toward an individualized target-based approach. For the first time, older and unfit younger patients could be treated with less intensive protocols but encouraging results. A schematic representation of the present genomic classification of AML is shown in Table 1. It must be underlined that both classifications fit patients younger than 65 years.

The prognostic value of genomic classification is less reliable in older patients and in therapy-related AMLs. Moreover, it has the best value in intermediate cytogenetics: in the presence of an adverse karyotype, cytogenetics generally supersedes mutations, while in the presence of favorable cytogenetics, adverse molecular mutations may lose their unfavorable significance. Many factors influence the prognostic significance of mutations, such as the size of mutated clones, the co-occurring mutations, and the employed therapy. So, the current classification is likely to evolve after large-scale analysis in different subgroups.

The definition of different molecular-based AML subgroups has led to the development of different target therapies. Since 2017, nine new drugs have obtained FDA approval for AML treatment and were quickly included in standard protocols, thus changing the conventional therapy landscape of adult and pediatric AMLs. However, despite the riches of genetically-driven protocols and an initial advantage in CR rates, long-term prognosis remains dismal, as the cumulative 5-years OS is still around 50% in younger patients and 25% in those aged 60 years or more [24]. These data suggest that the current genomic classifications that mask or overlap leukemia subgroups are not completely defined, but also evidences that the addition of target drugs has, to date, failed to eradicate leukemia stem cells (LSC).

The existence of a hierarchical organization of a leukemic population resembling the normal hematopoietic model and the presence of a small cohort of leukemic cell with stemness properties, sharing the bone marrow niche with the normal counterpart, was first hypothesized by Fialkow in 1978 [25,26] and definitively proven two decade later in SCID mice transplant models [27,28]. For their capacity to acquire transient quiescence and dormancy, as well as resistance to DNA damage, LSCs are considered “intrinsically resistant” to conventional antiproliferative therapies [29,30]. However, more recent studies demonstrated that under chemotherapy pressure, other mechanisms can induce resistance, not necessarily involving only LSCs. Guryanova et al. demonstrated that the acquisition of *DNMT3A* mutations seems to be able to drive resistance to anthracycline-based chemotherapy as the result of a defect of nucleosome remodeling that affects DNA damage response [31]. Farge et al. observed a survival advantage in leukemia cells with high oxidative phosphorylation (OXPHOS) status, irrespective of stemness status [32]. Duy et al. showed that leukemia cells surviving chemotherapy acquire a transient senescence-like phenotype, with high proliferative and engraftment potential, responsible for relapse, again irrespective of their stemness [33]. Altogether, these data show that the LSC landscape is largely affected by chemotherapy, which shapes LSC status by inducing patient-specific, dynamic resistance properties, making them difficult to target a distinct LSC state. Despite phenotypic plasticity also affecting the expression surface markers, the changes are expected to be transient and reversible; thus, targeting surface antigens appears a promising approach to eradicate LSC clones.

Although it is well known that LCSs are enriched within the CD34+CD38- fraction, a clear definition of the LCSs phenotype does not exist. Compared to normal HSCs, LCSs may have higher expression of CD25, CD32, CD44, CD96, CD123, CD200, GPR56, N-cadherin, Tie2, TIM-3, CLL-1, c-MPL, and HDM2 [34,35,36], all representing potential targets for monoclonal antibodies (MAbs)-based immunotherapy. Over the past three decades, MAbs have revolutionized the prognosis in lymphoma patients and the recent availability of antibodies targeting neoplastic plasma cells will likely repeat this success in multiple myeloma. In AML, the scenario appears to be complicated by the low antigen expression of target molecules, by its variable expression during disease course, and by the difficulty in identifying surface markers exclusively expressed by leukemia cells. As such, to date, the use of MAbs in AML obtained less enthusiastic results.

In this paper, we will summarize the rationale, the clinical data, and the toxicity profile of immune-based therapies in AML, focusing on membrane antigens preferentially expressed on leukemia cells. Antibodies targeting immune checkpoints or cell therapy with lymphocytes charged with chimeric antigen receptors (CAR) against myeloid leukemia antigens are beyond the scope of this work.

## 2. MAbs in Cancer Therapy: Background and Rationale

The development of the hybridoma technology by Milstein and Kohler in 1975 [37] allowed the production and selection of highly specific mouse antibodies targeting human antigens, sometimes able to discriminate between antigen differing by a single amino-acid or for a post-translational modification [38,39] and thereby potentially enlarging the therapeutic windows of conventional chemo- and radiotherapy. Unfortunately, the infusion of mouse antibodies in humans generates an anti-antibody immune response, resulting in a loss of efficacy and increased toxicity [40]. Consequently, all of them were withdrawn after regulatory approval, with the exception of blinatumomab, a bispecific antibody targeting CD19 that uses two mouse-derived single chain variable fragment and lacks Fc segment and that is currently used for the treatment of acute lymphoblastic leukemia (ALL) in adults and children [41,42]. The technology progress led to the production of chimeric antibodies, obtained by binding the mouse variable region with the human immunoglobulin heavy chain and human kappa light constant region [43,44], still potentially recognized as foreign [45], and finally to the generation of humanized and fully human antibodies [46]. A large analysis of clinical studies with various antibody constructs showed a progressive reduction in immunogenicity using humanized or human antibodies compared to chimeric [40], and at present, antibodies entering clinical trials are either humanized or fully human products.

**Figure 1:**
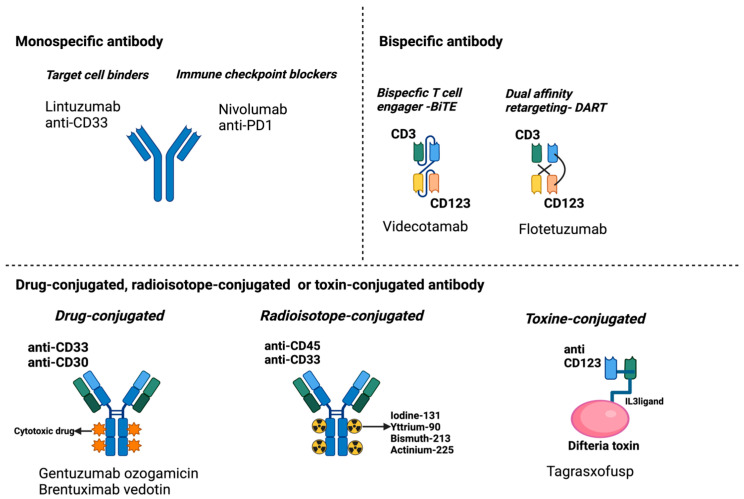
Antibody formats in clinical trials for AML. Created in BioRender.

Different antibody formats are in use for cancer therapy, differing in their structure and mechanism of action: monospecific antibodies, bispecific antibodies, and payloads conjugated antibodies (such as drugs, toxins, or radioactive isotopes). A schematic representation of the different antibody structures is shown in Figure 1.

### 2.1. Monospecific Antibodies

The most common isotypes for therapeutic purpose are IgG1 and IgG4, which are used to take advantage of their long half-life, permitting administrations every 21 days [47,48]. More recent strategies for MAbs optimization include molecular engineering aimed to prolong their half-life [49], to enhance target affinity [50] or effector function [51], and to increase their delivery at the target site, preventing aggregation [52]. In solid tumors, monospecific antibodies led to cancer cell death by directly blocking the survival signals from growth factors and by blocking angiogenesis [53]. In hematologic malignancies, they allow cell death through recruitment and activation of immune effector cells (T-cells of NK cells). The engagement of Fc Receptor (FcR) on effector cells results in antibody-dependent cellular cytotoxicity (ADCC) and/or antibody-dependent cellular phagocytosis (ADCP) [54]. Moreover, the IgG1 subclass can also bind the protein C1q, generated by complement activation, inducing cell death by complement-dependent cytotoxicity [55]. To the family of monospecific antibodies belong the checkpoint inhibitors, molecules that target immune cell regulatory ligands, potentially reactivating lymphocytes’ anti-tumor activity [56]. The mechanism of action and efficacy in cancer immunotherapy is beyond the scope of this paper, but an exhaustive review of the current knowledge in cancer has been provided by Kong at al. [57] and Arafat et al. [58].

### 2.2. Conjugated Antibodies

These antibodies are linked either to cytotoxic drugs (ADCs), which are the most used, to immunotoxin from bacteria or plants, or to radioactive isotopes, which are still under investigation.

The binding of ADCs molecules to the tumor antigens leads to internalization of the complex antibody-antigen and to the release of cytotoxic drug inside the cell, avoiding off-target toxicity. Most ADCs use humanized or human IgG1 to maximize half-life and enhance ADCC and ADCP efficacy [59,60]. Moreover, crucial for optimal functioning are the choice of the tumor target, to assure on-site drug delivery, and the conjugation method of the linker, which can affect the drug/antibody ratio, or induce early release of the active drug, thus reducing its efficacy and enhancing systemic toxicity [61,62]. The active drug is usually a small molecule that induces cell cancer cell death through direct DNA damage, microtubule network disruption, or inhibition of topoisomerase activity [63,64]. The currently used drugs are active at nano-molar concentrations, such as the topoisomerase inhibitor DXD, or even at pico-molar concentrations, such as the DNA damaging agents pyrrolobenzodiazepines (PBDs). Despite being generally better tolerated than conventional drugs, many off-target toxicities have been reported in the clinical use, including infusion reactions, cytopenia, infections, gastrointestinal symptoms, and transaminitis [65].

In toxin or immunotoxin-conjugates, antibodies are linked to an active drug of bacterial or plant origin. The only immunotoxin conjugate approved by the FDA and EMA for the treatment of hairy cell leukemia, moxetumomab pasudotox, in which a CD22 binding Fv was combined with truncated Pseudomonas exotoxin A, was stopped in 2023 due to low clinical activity [66]. In general, immunotoxin conjugates have a high immunogenicity and severe toxicities, such as capillary like syndrome (CLS) and hemolytic-uremic syndrome (HUS), that limit their use [66].

In radioisotope conjugates, the antibody targeting a neoplastic antigen is linked to a radioisotope, emitting beta particles that cause DNA-strand breaks and cell death, and do not require cell internalization [67]. The limited number of approvals and the low clinical use probably depend on logistical reasons and the need for a multidisciplinary team, which is not always available [68].

### 2.3. Bispecific Antibodies

**Figure 2:**
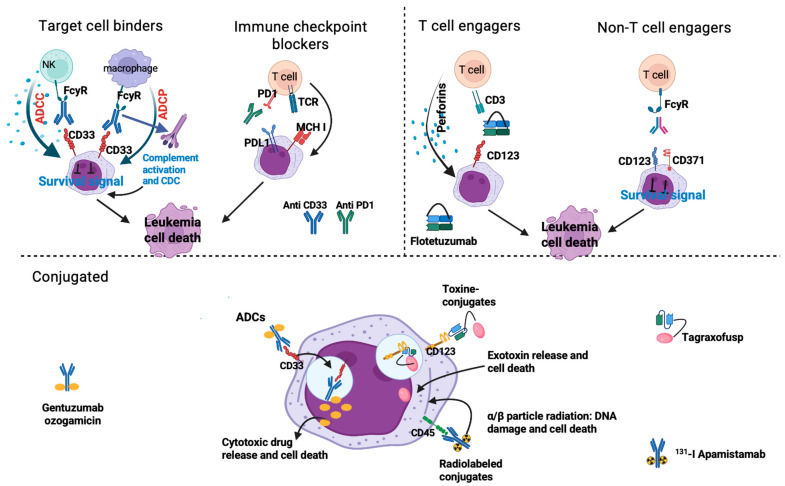
Mechanisms of action of monoclonal antibodies against leukemia cells. Created in BioRender.

Bispecific antibodies can recognize two different antigens or epitopes, localized on the same cells or on different cell types. Bispecific antibodies targeting two different cells are mostly T cell engagers (BiTEs), redirecting T effector cells to kill cancer cells after binding. The second class of bispecific antibodies engages two different antigens of cancer cells and induces cell death by blocking proliferation signals and by activating NK and macrophages [69]. Initially they were generated by chemical conjugation of two different purified MAbs or by fusing two hybridomas [70]. More recently, genetic engineering has generated over 50 different formats, revolutionizing the development of bispecific antibodies and allowing us to adjust their size, valency, and half-life to maximize efficacy [71]. In general, bispecific antibodies can be divided into two major classes: bearing FcR or lacking FcR. The presence of FcR contributes to antibody solubility and stability, prolonging half-life and assuring Fc-mediated effector functions such as ADCC and CDC [72]. In bispecific antibodies lacking FcR, therapeutic activity depends only on their antigen-binding capacity. Although the small size might favor tissue distribution and tumor penetration, it also accelerates renal elimination and reduces tissue concentration, thereby affecting the frequency of infusions [73]. With this premise, the ideal uses of antibody fragments are as carries of cytotoxic drugs and in T cell engagers constructed to manage the cytokine release syndrome (CRS) and immune effector cell associated neurotoxicity syndrome (ICANS), frequently occurring as the consequence of the sudden immune activation caused by bispecific antibodies [74]. A schematic representation of mechanisms of action of monoclonal antibodies according to their construction is shown in Figure 2.

## 3. MAbs in AML

The major challenge for MAbs use in AML is the identification of a suitable ligand. The ideal target for immunotherapy should be highly expressed on the majority of leukemic cells, sparing the normal hematopoietic counterpart or other normal tissues, to avoid off-target toxicity and allow recovery of the normal hematopoiesis [34]. Moreover, its expression should be stable during disease course, and it possibly should be involved in the leukemogenesis pathway to induce a survival disadvantage in targeted leukemia cells.

**Figure 3:**
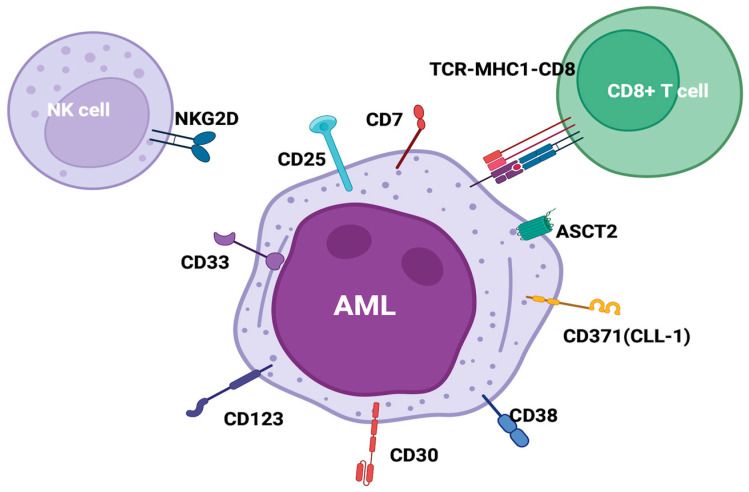
AML/LSC target antigen under investigation in clinical trials. Created in BioRender.

Unfortunately, compared to lymphoid diseases, AML is genetically more heterogeneous and the driver oncogenic lesions vary within and among patients [75]. Furthermore, the AML mutational burden, thus the generation of new potential leukemia-specific antigens, is fifty times lower compared to solid tumors, and new proteins resulting from chromosomal translocations or from gene mutations during leukemogenesis are poorly immunogenic or not expressed on cell surface [75,76]. With this limitation, clinical studies focused on CD33, CD123, and CD371 (CLL-1/Cec12), which show sufficient differential expression on AML and hematopoietic cells, and other potential targets, such as CD38, FLT3, CD56, CD30, CD7, and CD25, are under investigation (Figure 3).

### 3.1. Unconjugated Antibodies

#### 3.1.1. Anti CD33

CD33 is a member of sialic acid-binding immunoglobulin-like lectins (Siglecs) expressed on early myeloid progenitors and in over 90% of AML blasts [77]; its high expression has been associated with poor clinical outcome [78].

Early clinical trials with lintuzumab (SGN-333, HuM195) as a single agent demonstrated promising activity and good tolerability [79]. However, subsequent clinical trials investigating lintuzumab in combination with cytarabine od intensive therapy (mitoxantrone, cytarabine, etoposide) or as maintenance chemotherapy in relapsed or refractory (R/R) AML (NCT00002609, NCT 00002800, NCT 00006084, NCT 000016159, NCT0000283114, NCT 00502112, NCT00528333, NCT00997243) failed to demonstrate a survival advantage [80,81], leading to discontinuation of further development.

Despite the initial disappointing results with unconjugated anti-CD33 antibodies, a new fully humanized, unconjugated agent (BI-836858) with improved FcR aimed at NK-mediated ADCC has been developed. In early studies, it demonstrated higher blast cell lysis after combination with decitabine, which induced the upregulation of NKG2D ligand in leukemic cells boosting the NK mediated killing activity [82]. A phase I/II clinical trial (NCT02632721) exploring the maximum tolerated dose, safety, and efficacy in R/R AMLs has been recently concluded. The study enrolled 35 adult patients (age: 52–75 yrs) with an overall response rate (ORR, i.e., CR+CRi) ranging from 50% in patients receiving lower doses to 66% in those treated with higher doses of the drug. Adverse events included infusion reactions, bleeding, infections, and increased creatinine. However, the sponsor stopped the development of the drug and a phase II study was not performed.

#### 3.1.2. Anti CD123 Antibody

CD123 antigen (IL-3R) expression in AML is associated with a high proliferation rate and poor prognosis [83]. The first anti-CD123 antibody (talacotuzumab or CSL360) was a recombinant chimeric immunoglobulin G1, preventing the IL3 binding to its receptor. The antibody was tested in a phase I, multicenter study (NCT 00401739) enrolling 26 R/R AML cases. Despite a good tolerability, clinical response was low (1 CR and 1 PR) and monoclonal blockade of CD123 function was considered insufficient, resulting in cessation of further development [84]. A second generation of anti-CD123 antibody (CSL362), fully humanized and with FcR engineered to increased binding to NK cells through Fcy receptors, demonstrated better efficacy and tolerability, with only 3 severe adverse events among 25 patients in a phase I trial (NCT 0162852) involving patients in first or second CR. Ten out of twenty-five patients maintained CR for over six months and three out of six patients reached MRD negativity [85]. However, in a phase II trial, talacotuzumab plus decitabine failed to show any clinical benefit compared to decitabine alone in older patients with newly diagnosed AML [86], and its further development was halted.

#### 3.1.3. Anti CD38 (Daratumumab, Isatuximab)

CD38 is a transmembrane glycoprotein acting as an adhesion partner for CD31 or as an ectoenzyme involved in the catabolism of NAD+ and NADP. CD38 is largely expressed on neoplastic plasma cells, but also in myeloid and erythroid precursors, as well as in over 70% of AML cases [87], thus representing a potential target for MAbs therapy. In vitro studies demonstrated that targeting CD38 on AML by a blocking antibody facilitates leukemic cell removal by immune cells and inhibits leukemic blast metabolism, impeding mitochondrial transfer from mesenchymal to leukemic cells [88], providing a rationale for its use in AML. A phase I/II trial of daratumumab in R/R AML and in high-risk MDS (NCT03067571) has been terminated, but results are not yet available.

Another anti CD38 antibody, isatuximab, was studied in a phase I/II trial (NCT03860844) enrolling 27 AML patients in first or second relapse. Preliminary results showed an ORR of 65.2% and a median OS of 9.4 months. Serious adverse events were experienced in 62.9% of patients, including anemia, febrile neutropenia, epistaxis, infections, gastrointestinal disorders, headache, and skin rash. The study was stopped by the sponsor because efficacy criteria were not met, not for safety concerns.

#### 3.1.4. Anti FLT3

FLT3 (FMS-like tyrosine kinase 3), a member of the class III receptor tyrosine kinase family, is highly expressed in the blasts of both AML and ALL. FLT3 plays an important but not exclusive role in maintaining the survival of normal HSCs, and its recurrent mutations, either as internal tandem duplications (ITD) or in the tyrosine kinase domain (TDK), are found in many AML cases [89,90]. Animal transplant models with non-functioning and wild-type FLT3 showed that hematopoiesis is almost normal in FLT3 knockout animals, while FLT3 mutations give a significant growth advantage, suggesting that selective FLT3 inhibition can block excessive activation of leukemic blasts with acceptable hematopoietic toxicity [90]. On this basis, an anti-FLT3 MAb (LY3012218) was developed and evaluated in a phase I trial in R/R AML (NCT008879269), but failed to demonstrate clinical efficacy; the failure was attributed to the high leukemia burden and to the non-optimized FcR, which was unable to activate ADCC [91]. Subsequently, a Fc optimized anti-FLT3 antibody (FLYSIN) was evaluated as a single agent in a phase I trial in a cohort of MDR+ AMLs. An antibody dose dependent reduction in bone marrow MRD was observed in 20/31 patients (65%), and in 2 patients, MRD negativity was reached. The beneficial effect, the favorable toxicity profile, and the absence anti FLYSIN autoimmunity despite its chimeric structure, justify the investigation of the potential synergistic effect with other agents, such as hypomethylating agents, for the eradication of MRD [92].

### 3.2. Conjugated MAbs (ADCs)

#### 3.2.1. Anti-CD33—Gemtuzumab Ozogamicin

Gemtuzumab ozogamicin (GO) was the first ADC approved by the FDA in 2000 for the treatment of older patients with CD33 positive AML in first relapse. The drug, used as monotherapy at a dose of 9 mg/sqm repeated every 14 days for 2 doses, showed an ORR of 30% in patients over 60 years with relapsed AML [93]. However, a post-approval phase II trial (SWOG S0106) adding GO (6 mg/sqm) to standard induction chemotherapy in younger patients did not confirm the results obtained in elderly patients [94]. In addition, a higher induction mortality was observed in patients receiving GO compared to those treated with standard chemotherapy (5.5% vs. 1.4%), resulting in the voluntary withdrawal of the drug from US market in 2010. Concerns were related to the higher incidence of veno-occlusive disease (VOD), being 9–14% in patients receiving GO in combination with hepatotoxic agents or within 3 months from allogeneic HCT [95]. This was ascribed to the direct toxicity of calicheamicin on hepatocytes, or from the uptake of GO by CD33+ cells residing in the hepatic sinusoids [95,96]. Since then, several other phase III studies evaluated the efficacy of GO in frontline treatment of adult AML, using different doses and schedules (Table 2). In particular, the ALFA-0701 trial (NCT 00927498) employed fractionated and lower doses of GO in patients aged 50 to 70 years, demonstrating a favorable safety profile and a better EFS in patients receiving GO [97], paving the way for GO reapproval in 2017.

A systematic metanalysis including 3325 patients from randomized and open label trials with survival as an endpoint confirmed the safety profile of a reduced dose (3 mg/sqm) and the survival advantage in patients receiving GO, irrespective of age, in favorable and intermediate cytogenetic groups, concluding that a single dose of GO can be safely added to conventional therapy [102]. Freeman et al. have recently demonstrated the benefit of a second dose of GO at day +4 of induction therapy, observing a greater benefit on MRD and improved OS in older patients without adverse cytogenetics [103]. Xu et al. conducted a metanalysis of 15 randomized clinical trials and 15 retrospective trials to test the clinical benefit and safety of GO in different leukemia subgroups, confirming the survival advantage in favorable and intermediate cytogenetics, but also in NPM1 mutated and in FLT3-wt AMLs and in patients aged < 70 years [104]. Fournier et al. retrospectively investigated the predicting value of the genetic background and co-occurring mutations on the benefit of the addition of GO to standard front-line chemotherapy. Using the ELN classification, they confirmed that the benefit of GO was restricted to favorable and intermediate risk categories, especially among those bearing activating signaling mutations, which correlated with higher CD33 expression [105].

Ganzel et al. reported the feasibility of the addition of GO to CPX-351 in favorable/intermediate risk, newly diagnosed FLT3-ITD negative AML [106]. Estey et al. observed higher response rates and superior RFS with the addition of GO to ATRA in acute promyelocytic leukemia (APL) [107]. Ravandi et al. showed a 5-year EFS of 84% through the addition of GO to arsenic trioxide (ATO) and ATRA in high risk APL, and suggested that GO combination could potentially eliminate the need for anthracycline in these patients [108]. Many other trials investigating other chemotherapy combinations or disease subgroups are currently ongoing or have recently concluded (Table 3). Details on AML subgroups and endpoints are available at https://www.clinicaltrials.gov.

#### 3.2.2. Other Anti-CD33 ADCs

Vadastuximab (SGN33A) is an anti-CD33 antibody linked to a synthetic pyrrolobenzodiazepine dimer (PBD), a potent compound producing DNA damage and cell cycle arrest with consequent leukemia cell apoptosis. In preclinical studies, SGN33A have demonstrated higher activity than GO in AML cell lines and in leukemia cells, regardless of cytogenetic status [109]. A phase I trial assessing the safety and activity of vadastuximab alone or in combination with hypomethylating agents (HMAs) demonstrated high ORR in relapsed (28%) and in newly diagnosed AML (73%), with 47% of responding patients achieving also MRD negativity [110,111]. Despite a good tolerability, patients treated with SGN33A experienced severe myelosuppression [110,111]. An increased incidence of infection deaths in the experimental arm led to the conclusion of the confirmatory phase III trial (CASCADE) comparing HMAs with or without vadastuximab in untreated AMLs [112].

IMGN779 is an anti CD33 antibody linked to an alkylating drug (indolinobenzodiazepine-pseudodimer IGN) with potent anti AML activity in vitro, mostly in the presence of FLT3-ITD [113]. In long term cultures, it reduced leukemia colonies in a dose-dependent manner without affecting normal hematopoietic cells [114]. The preliminary results of the NCT02674763 phase I trial, in R/R CD33+ AML, reported good tolerability and safety, with a response over 50% in patients receiving weekly administration and 35% in those treated every second week [115].

#### 3.2.3. Anti-CD123

Tagraxofusp (SL401, TAG) is an anti-CD123 recombinant fusion protein composed by the truncated diphtheria toxin and a human IL-3 ligand. After ligand, the compound is internalized and induces cell death through inhibition of protein synthesis [116]. TAG received FDA approval for the treatment of blastic plasmocytoid dendritic cell neoplasm (BPDN). A phase I clinical trial (NCT03113643) investigated TAG in combination with azacytidine (aza) and venetoclax (ven) in high-risk AML: CR was obtained in 39% and CRi in 12% of enrolled patients, and 71% obtained MDR negativity. Of note, among 13 patients with TP53 mutation, 54% responded favorably [117]. The safety and efficacy of the TAG-aza-ven combination encourages its use in the frontline setting, including those with high risk genetic mutations, including TP53.

Pivekemab sunirine (IMGN632) is an anti-CD123 antibody, bound to a DNA alkylating drug (indolinobenzodiazepine pesudodimer). In a phase I/II clinical trial (NCT04086264) in R/R AML, IMGN632 showed a safe profile and good tolerability. Preliminary results of phase II part evidenced a CR rate of 17%, which led to a phase I/II study exploring the combination with aza and ven [118].

#### 3.2.4. Anti-CD371 (CLL1, Clec12A)

CLL1 is a transmembrane glycoprotein overexpressed in myeloid leukemia cells and in LSCs. Its function, as well as its ligand, are not completely understood. It has been suggested that it is involved in regulating some inflammatory situations by down modulating granulocyte and monocyte function [119]. Being selectively expressed on LSCs, even more than CD123, which is present also in normal progenitors, CLL-1 could be regarded as an ideal target for anti LSCs immune therapies [120,121]. A phase I dose escalation trial (NCT03298516) investigated the DCLL9718S, a humanized antibody targeting CLL1 linked to PBD, in R/R ALML, either as a single agent or in combination with aza, in previously untreated AML unsuitable for intensive therapy. Among the 18 patients receiving DCLL9718S, none obtained CR or PR. The study was stopped during the dose escalation phase based on assessment of safety and efficacy and will not move to phase II [122].

#### 3.2.5. Anti-FLT3

The phase I trial (NCT02864290) testing AGS62P1, an anti-FLT3 antibody-amberstatin 269 (an microtubule disrupting agent), in R/R adult AML has been closed for lack of efficacy [123]. Able et al. have recently published the promising results of preclinical data on two anti FLT3-20D9h3 ADC delivering the DNA-alkylator duocarmycin (DUBA) or the microtubule-toxin monomethyl auristatin F (MMAF). In vitro assays found that FLT3-20D9h3-DUBA completely eliminated leukemia progenitor cells and prevents engraftment of patients-derived xenograft cells (PDX) in NSG mice. The lack of toxicity on healthy hematopoietic cells provides the rationale for anti FLT3-ADCs as a therapeutic LSC target [124].

#### 3.2.6. Anti-CD25

The human CD25 antigen is the alpha-chain of the heterotrimeric interleukin-2 receptor, a key component in regulating normal immune function [125], and its expression is normally limited to activated T and B cells and regulatory T cells (Tregs) [125]. Cell surface expression of CD25 on AML and ALL blast cells is associated with failure of induction therapy, increased risk of relapse, and shorter OS [126,127]. With this background, the safety, activity pharmacokinetics, and immunogenicity of camidanlumab tesirine, an anti-CD25 antibody conjugated with PBD, were investigated in a phase I, open-label, dose escalation, and expansion study (NCT02588092). Among 34 highly pretreated, R/R CD25+ AML patients, 2 achieved CRi. Therapy showed good tolerability, and no serious adverse events were registered. The study was stopped for programmatic reasons before the individuation of recommended dose [128].

#### 3.2.7. Anti CD30

Brentuximab vedotin (BV) is an antibody-drug conjugate consisting of a human anti-CD30 antibody covalently attached to the antimicrotubule agent monomethyl auristatin E (MMAE) through a protease-cleavable linker [129]. After binding the cell receptor, the complex CD30-BV is internalized and the MMAE released by proteolytic cleavage interacts with tubulin inducing cell cycle arrest and apoptotic death [129]. BV is currently approved for Hodgkin lymphoma and anaplastic large cell lymphoma. However, an aberrant expression of CD30 has been reported in AML, making anti CD30-BV conjugate a potential target for the treatment of AML. [130,131]. Narayan et al. have explored the activity of BV in combination with MEC (mitoxantrone, etoposide, cytarabine) in a phase I trial for relapsed/refractory AMLs (NCT01830777). Among the 22 enrolled patients, the composite response rate (CR+CRi) was 36%, with a median OS of 9.5 months and a median disease-free survival of 6.8 months in responders. Approximatively 55% of patients were able to proceed with allogeneic stem cell transplantation or donor lymphocyte infusion. The side effects profile was similar to that of MEC alone. The authors concluded that the combination of BV with MEC is safe, and future randomized trials are warranted to compare BV this combination with MEC in R/R AMLs and to discover possible differences among cytogenetic/molecular subgroups [132].

#### 3.2.8. Anti ASCT2

The sodium-dependent alanine-serine-cysteine transporter 2 (ASCT2), also known as SLC1A5, is a member of the solute carrier 1A (SLC1A) family, and is preferentially involved in transport of glutamine across the cell membrane [133]. ASCT2- overexpression has been reported in solid cancers, where it is associated with poor prognosis [134], and in hematologic malignancies, including AML [135]. The efficacy of the first in class anti ASCT2 antibody conjugated with a pyrrolobenzodiazepine dimer (MEDI7247) was tested in a phase I trial (NCT03106428) for R/R AML, diffuse large B cell lymphoma, and multiple myeloma. ORR was 22% in the AML cohort, including CRi (one patient) and MLFS (five patients). Toxicity was mostly hematologic, with grade 3/4 cytopenia in 37% of AML patients, and incidence increased after repeated doses, so the study was terminated early due to the limited efficacy, and any further development of MEDI7274 for R/R hematologic malignancies has been not supported. However, the authors concluded that other strategies to improve the therapeutic index of anti-ASCT2 conjugates is warranted [136].

#### 3.2.9. Radio-Conjugated Antibodies

Radioimmunotherapy (RIT) utilizes MAbs labeled with radio nuclides, assuring continuous radiation exposure to cells expressing target antigens. RIT can deliver ionizing radiation to disease sites more specifically than traditional total body irradiation. Ionizing radiations delivered to AML cells induce cell death by two main mechanisms, apoptosis and necrosis, as the result of unrepaired DNA breaks [137]. The nature of radiation emitted (α or β) depends on the used radio nuclide. In AML, the most used emitters have been ^131^I (iodine 131) or ^90^Y (Yttrium 90), both β emitters with lower linear energy transfer (LET) and relative biological effectiveness (BE) compared to α-emitters, but deemed safer and more manageable for healthcare professionals [138]. Radiolabeled antibodies were investigated mostly in the setting of preparative regimens for allogeneic HCT, with the aim to reduce overall toxicity while maintaining immunomodulation to permit engraftment and to reduce leukemia burden [139,140,141,142,143]. The most used cell targets are CD45 and CD33, highly expressed in AML and with diffuse expression in hematopoietic compartment. Gyurkocza et al. have recently published the result of a phase III SIERRA trial (NCT 02665065) comparing a targeted pretransplant regimen involving the anti-CD45 radio-conjugate ^131^ I- apamistamab with conventional conditioning in elderly patients (median age 64 years) with R/R AML. The study demonstrated that the target regimen was associated with higher durable CR rate, and was well tolerated in heavily pretreated patients [144]. More recently, a sequential combination of anti-CD33 MAb linked to an α-emitter (^213^Bi-lintuzumab) and cytarabine was investigated in patients with advanced AML in a phase I/II trial (NCT00014495). A reduction of bone marrow blasts was observed in 70% of patients and a clinical response in 24%, with an ORR of 19% and with acceptable toxicity, providing the rationale for the use of RIT with α-emitters as a salvage therapy [145]. The combination of fractionated doses of ^225^ AC-lintuzumab and cytarabine was then studied in 40 elderly patients with untreated AML, confirming its antileukemic effect and safety [146]. Many studies investigating α-radionuclides-conjugate antibodies as a part of reduced intensity conditioning regimens and in different donor settings are still ongoing (Table 4). The information from all these trials will provide a guidance for further clinical implementation aimed toward an individualized treatment in line with the emerging “theranostics” concepts.

### 3.3. Bispecific Antibodies

The promising results obtained by antiCD19/CD3 blinatumomab in ALL provided the scientific rationale to further explore bispecific antibodies in AML [147]. However, early trials using antibodies targeting CD33, CD123, and CLL1 showed limited efficacy and were terminated early [148,149]. Recent advances in engineering technologies have led to new generations of bispecific antibodies with encouraging early results. A summary of published studies on bispecific antibodies in AML is reported in Table 5. Seven of the nine published trials investigated bispecific antibodies targeting CD33 and CD123. In the last two, targets were CD38 and a novel surviving protein [150,151]. The majority redirect T cell via CD3, and one promotes NK activation via CD16 and interleukin-15 (TriKE) [152].

## 4. Conclusions and Future Perspectives

Despite robust in vitro evidence and the technological progress that permitted the production of highly sophisticated antibody-based therapies, at present, the only immune therapy with proven efficacy that is routinary used in AML clinical care is still GO. The addition of GO to conventional induction chemotherapy granted a survival benefit for patients with favorable and intermediate risk AML, but not in those with adverse cytogenetics or molecular features. The major obstacle to the success of antibody therapy in AML is disease heterogeneity and its low mutational burden compared to other tumors, which account for the difficulty in identifying targets exclusively expressed on LSCs. In addition, the knowledge of the full complement of antigens expressed on mature and stem leukemic populations is not complete, and it is partly limited by study design, mostly based on transcriptomic analyses that do not consider the high translational control of gene expression.

Bordeleau et al. have recently proposed a surface proteomic analysis to identify potential targets for immunotherapeutic intervention, uncovering 30 potential AML-specific leukemia stem cell markers [159], the intensity expression and stability along the disease course of which is far from being established. The next step will be the use of surfaceome analysis to identify the antigens expressed in distinct molecular subgroups, paving the way to a genomic-driven antibody approach. Besides the identification of the ideal, though uncommon, leukemia-specific targets, future efforts should be focused on planning strategies that boost antibody selectivity, which represents the key to ensuring the safety and efficacy of immune therapy targeting the more common but less specific leukemia-associated antigens. The generation of dual/multi-specific antibodies targeting leukemia-associated antigen combinations may enhance leukemic cells killing while sparing the normal counterpart. A further increase in selectively and efficacy might result from antibody engineering to apply an “AND-gated strategy”, permitting antibody activation only if all its targets are engaged. Another method to increase antibody selectivity could rely on the modulation of valency. Designing antibodies with multiple binding sites for AML blast antigens, as well multiepitope antibodies, may increase their killing capacity, mainly in cases of low target antigen expression.

The other crucial future challenge will be to engineer the Fc fragment to reinforce effector functions and prolong antibody half-life, circumventing the need for frequent administration and, especially for ADCs, to increase antibody therapeutic index. Co-administration of unconjugated and drug-conjugated antibodies could increase systemic antibody exposure and lower the drug-antibody ratio, limiting the toxicity on normal tissues and enhancing ADC efficacy. Moreover, the improvement of cytotoxic drug linkers and the use of drugs with high cytotoxicity, short half-life, and incomplete hydrophobicity to avoid antibody aggregation will improve ADCs safety.

Last, efforts should focus on the reversion of the immunosuppressive AML microenvironment. In this instance, bispecific antibodies represent a promising off-the-shelf strategy, potentially superior even to CAR-T therapy. The aim could be to engineer constructs to prolong half-life without increasing on-target off-tumor toxicity, such as CRS and ICANS, the duration and severity of which can be affected by slow antibody clearance. A role in the disappointing results in early clinical trials with bispecific antibodies was certainly due to disease phase. On-target-based resistance, as well pharmacokinetics and T cell exhaustion and antigen loss, can hinder bispecific antibody efficacy. However, the high leukemic burden itself represents a premise for bispecific antibody treatment failure. A recent study employed a mathematical model to investigate leukemia cell kinetics in the presence of T cells and bispecific antibodies, demonstrating that the efficacy of bispecific antibodies is largely influenced by leukemia burden, irrespective of T cell activation and killing ability [160], suggesting three options to enhance treatment: by maintaining high T cell levels, by reducing leukemia burden, or by assuring a high antibody concentration through increased infusion frequency and/or dosage. None of the three proposed solutions is easily applicable in clinical practice, but a better allocation of bispecific antibodies during treatment can contribute to enhancing their potentiality.

Despite the slow start and pace, we are confident that immune therapy will find the right place in AML treatment, favored by rapid technologic progress, which will help in developing more efficient therapeutic tools and to combine different approaches with synergistic effects.

## Figures and Tables

**Table 1:** Genomic classification of AML according to European Leukemia Net (ELN) and National cancer Centers Network (NCCN).

**1. ELN**		
**Risk**	**Genetic lesions**	
Favorable	t(8;21); RUNX1::RUNX1T1	
	inv(16)(p13.1q22); CBFB::MYH11	
	Mutated NPM1 without FLT3-ITD bZIP in-frame mutated CEPBA	
Intermediate	Mutated NPM1 with FLT3-ITD Wild type NPM1 with FLT3-ITD t(9;11)(p21q23.3); MLLT3::KMT2A Cytogenetics abnormalities not classified as favorable or adverse	
Adverse	t(6;9)(p23;q34.1); DEK::NUP214	
	t(v;11q23.3);KMT2A rearranged	
	t(9:22)(q34.1); q11.2); BCR-ABL	
	inv (3)(q21.3q26.2) or t(3;3)(q21.3;q26.2); MECOM (EVI1) -5 or del(5q); -7; -17/abnormality (17p) Complex karyotype (≥3), Monosomal karyotype Mutated ASXL1, BCOR, EZH2, RUNX1, SF3B1, SRSF2, STAG2, U2AF1, ZRSF2 Mutated TP53	
**2. NCCN**		
**Risk**	**Cytogenetics**	**Molecular abnormalities**
Better	Inversion (16) or t(16;16) t(8;21) t(15;17)	Normal cytogenetics: NPM1 mutation without FLT3-ITD; bZIP in frame CEPBA mutation
Intermediate	Normal cytogenetics Trisomy 8alone t(9;11) Other not defined	Mutated -NPM1 and FLT3-ITD Wild type NPM1 and wild type FLT3
Poor	Complex (≥3 clonal chromosomal abn) Monosomal karyotype -5; 5q-;-7; 7q- -11q23-non translocation (9;11) inv(3), t(3;3) t(6;9) or t(9;22) or t(8;16)	Mutated TP53 Mutated RUNX1, ASXL1, BCOR, EZH2, SF3B1,SRSF2, STAG2, U2AF11nad/or ZRSR2 Wild type NPM1 and FLT3-ITD (high allelic ratio)

Adapted from [1] and NCCN Version3.2024. https://www.nccn.org.

**Table 2:** Results and hepatotoxicity in phase 3 clinical trials including anti CD33-GO in the frontline treatment of adults AMLs.

Trial	Patients (N)	Age (Range)	GO Dose/Schedule	CR (%)	RFS	OS	VOD/SOS (N)/ ≥3 Hepatic Toxicity (%)	Ref
SWOG S0106	595	18–60	6 mg/m^2^ on D4	75	5-yrs 43%	5-yrs 46%	0/NA	[94]
GOLEAMS Aml 2006	254	18–60	6 mg/m^2^ on D1	92	3-yrs 51%	3-yrs 53%	4/23	[98]
MRC AML15	1099	18–59	3 mg/m^2^ on D1	82	5-yrs 39%	5-yrs 43%	0/NA	[99]
ALFA-0701	278	50–70	3 mg/m^2^ on D1,4,7	81	2-yrs 50%	2-yrs 53%	2/6	[97]
NCRI AML16	1115	51–84	3 mg/m^2^ on D1	62	3-yrs 21%	3-yrs 25%	0/17	[100]
GIMEMA AML19	237	61–75	6 mg/m^2^ on D1, 3 mg/m^2^ on D8, 2 mg/m^2^ monthly x8	15	1-yrs 10%	3-yrs 24%	0/7	[101]

GO: gentuzumab-ozogamicin; CR: complete remission; RFS: relapse-free survival; OS: overall survival; VOD: veno-occlusive disease; SOS: sinusoidal obstruction syndrome.

**Table 3:** Clinical trials investigating Gentuzumab-Ozogamycin (GO) in adult AMLs.

Trial Identifier	Title	Phase	Status
NCT03904251	CPX-351+GO in R/R AML	1	Completed
NCT04070768	Venetoclax+GO in R/R CD33 AML	1b	Completed
NCT03848754	Pracinostat+GO (PRAGO) in R/R AML	1	Completed
NCT00766116	Azacytidine+GO in R/R AML	1/2	Completed
NCT02182596	DNR+AraC and Fractioned GO in AML at first relapse	1/2	Completed
NCT03839446	Mitoxantrone, Etoposide and GO for AML refractory to Initial Standard Induction Therapy	2	Completed
NCT00044733	GO in AML relapsed after autologous or allogeneic SCT	2	Completed
NCT05558124	CPX-351 in combination with GO in newly diagnosed AML	1	Recruiting
NCT05716009	Tagraxofisp-erzs, an IL-3 Diphteria Fusion Protein in combination with GO in R/R AML	1	Recruiting
NCT04849910	Allogeneic engineered HCT lacking the CD33 protein, and post HCT treatment with GO, for CD33+Aml or MDS	1/2	Recruiting
NCT00801489	GO+Fluda+AraC+filgrastim-sndz+idarubicin in newly diagnosed AML and HR-MDS	2	Recruiting
NCT03672539	CPX-351 and GO in R/R AML or HRMDS	2	Recruiting
NCT04050280	Fractioned GO+Cladribrine +AraC+ G-CSF (CLAG-G) in persistent, R/RAML	2	Recruiting
NCT03737955	Fractioned GO in treating MRD in AML	2	Recruiting
NCT04168502	GO chemotherapy MRD levels; adult untreated, de novo favorable/intermediate risk AML	3	Recruiting
NCT03900949	GO and Midostaurin combination with standard Cytarabine and Daunorubi Midostaurin as a novel approach to treating patients with newly diagnosed FLT3 mutated AML	1	Active, NR
NCT04385290	Midostaurin +GO in first line standard therapy for Acute Myeloid Leukemia (MOSAIC)	1/2	Active, NR
NCT00658814	Azacitidine and GO in Treating Older Patients with previously untreated AML	2	Active, NR
NCT03374332	Fractioned GO followed by non-engraftment Donor Leukocyte infusion for R/R AML	2	Active, NR

GO: gemtuzumab-ozogamycin; AraC: cytarabine; DNR: daunorubicin; Fluda: Fludarabine; G-CSF: granulocyte-colony stimulating factor; IL-3: interleukin 3; FLT3: fms -related receptor tyrosine kinase 3; AML: acute myeloid leukemia; HR-MDS: high risk myelodysplastic syndrome; HCT: hematopoietic cell transplant; SCT: stem cell transplantation; R/R: relapsed/refractory; MRD: minimal residual disease.

**Table 4:** Ongoing clinical trials of RIT for AML with or without hematopoietic cell transplantation.

Target	Phase	Agent	Population	Donor	Trial Identifier
CD45	I/II	^211^-At-BC8B10 Fluda/Cy/2GyTBI	≥18 R/R AML	Haplo	NCT03670966
CD45	I/II	^211^-At-BC8B10 Fludarabine/TBI	18–75 (adult-older) AML, mixed AL, HR-MDS	HLA MRD or MUD	NCT03128034
CD33	I	^225^Ac-Lintuzumab+ CLAG-M	≥18 R/R AML	//	NCT03441048
CD33	I/II	^225^Ac-Lintuzumab+ Venetoclax	≥18 R/R AML (including secondary)	//	NCT03867682
CD25	I	^90-^Y-DOTA-Basiliximab Flu/Mel/TBI	18–60 high risk AML in CR or with sensitive disease	Allo-SCT	NCT05139004

^211^-At: astatine-221; 225Ac: Actinium-225; ^90-^Y-DOTA: Yttrio-90 DOTA; Fluda: Fludarabine; Cy: cyclophosphamide; Mel: melphalan; TBI: Total Body Irradiation; R/R: relapsed/refractory; AML: Acute myeloid leukemia; Mixed AL: Mixed phenotype acute leukemia; HR-MDS: high risk myelodysplastic syndrome; Haplo: haploidentical; MRD: matched related donor; MUD: matched unrelated donor.; allo-SCT: allogeneic stem cell transplantation.

**Table 5:** Results of Clinical trials with Bispecific antibodies in AML.

Antibody Construct	Target	Phase	Study Population	Response	Toxicity	Reference
BiTE	CD3xCD33	I	R/R highly treated	CR: 3/9 evaluable PR: 1/9 evaluable Blast reduction: 17/27 evaluable	Grade ≥3 febrile neutropenia (9/30, 30%) Grade 2 CRS (1/30) Therapy-related deaths: 0	[153]
	CD3xCD33	I	R/R adult AML	CR: 8/60 evaluable Blast reduction: 37%of non-responders	CRS: 78% (grade3/4: 10%) Infusion reaction: 30% Therapy-related deaths: 0	[154]
	CD3xCD33	I	R/R adult AML, MDS	No responders	≥1dose-limiting toxicity: 16% ≥1CRS: 42%, ≥ICANS: 7% Hematologic toxicity: 11% Therapy-related deaths: 16	[155]
	CD3xCD123	I	R/R AML, MDS	CR: 2/8 evaluable SD: 6/8 evaluable	CRS: 10%, ICANS: 10% Infusion reaction: 28% Therapy-related deaths: 0	[156]
	CD3xCD123	I	Adult de novo/secondary AML; B-ALL, BPDN, BP-CML	ORR (MLFS or better): 9%	≥1dose-limiting toxicity: 16/120 CRS: 59%, ICANS: 2.5% Hematologic toxicity: 51% Therapy-related deaths: 8	[157]
	CD3xCD38	I	R/R adult AML CD38+	MRD neg-CR:2 PR:2	Grade1/2 CRS: 62% Grade3/4 anemia: 8%, neutropenia: 15%, thrombocytopenia: 23% Therapy-related deaths: 0	[150]
	CD3xSurvivin peptide-targeting T-cell receptor	I	R/R Adult AML HLA-A2:01 restricted, NSCL cancer	PR: 1 AML	CRS: 13% ≥Grade3 AEs: 73% Therapy-related deaths: 0	[151]
DART	CD3xCD123	I/II	R/R Adult AML	CR: 26.7%, ORR 30% of 30 evaluable. mOS: 10.2 months	CRS 34%, grade ≥ 3 cytopenia 10%, peripheral edema 40.9%, Therapy-related deaths: 0	[158]
TriKE	CD16/IL15/CD33	I	R/R Adult CD33+AML, HR MDS	SD: 2	No toxicities reported Therapy-related deaths: 0	[152]

AML: acute myeloid leukemia, B-ALL: B acute lymphoblastic leukemia, BPDN: blastic plasmocytoid dendritic cell neoplasm, R/R: relapsed and or refractory, MDS: myelodysplastic syndrome, BiTE: bispecific T cell-engagers, TriKE: trispecific killer engagers, DART: dual affinity retargeting antibody, CR: complete remission, PR: partial response, SD: stable disease, MLFS: morphologic leukemia-free state, MRD: measurable residual disease, ORR: overall response rate, mOS: median overall survival, AE: adverse event, CRS: cytokine release syndrome, ICANS: immune effector cell neurotoxicity syndrome.
